# The clinical and pathological features of low-grade appendiceal mucinous neoplasm (LAMN)

**DOI:** 10.1007/s12672-026-04833-4

**Published:** 2026-04-02

**Authors:** Omar Hamdy, Gehad A. Saleh, Mona Hany Emile, Ahmed Elhadidy, Ahmed Ibrahim, Ola Elsayed, Ahmed Reda, Yasser Sharaf, Merna M. Hegazi, Osama Bahy, Mahmoud Soliman

**Affiliations:** 1 Surgical Oncology Department, Oncology Center, Mansoura University, Mansoura, Egypt; 2 Diagnostic and interventional radiology department, faculty of medicine, Mansoura University, Mansoura, Egypt; 3 Pathology department, faculty of medicine, Mansoura University, Mansoura, Egypt; 4 Mansoura University Hospitals, Mansoura, Egypt; 5 Royal Preston Hospital, NHS, Preston, UK; 6 Breast Surgery Department, North Manchester General Hospital, Manchester University Hospitals Foundation Trust, Manchester, UK

**Keywords:** LAMN, Pseudomyxoma peritonei, HIPEC, Appendiceal tumors

## Abstract

**Introduction:**

In this manuscript, we evaluated the clinicopathological features of low-grade appendiceal mucinous neoplasm (LAMN), a unique type of appendiceal tumors.

**Methods:**

This is a retrospective study that included patients with LAMN who were presented to our center from January 2008 to December 2024. The epidemiological, clinical, pathological, therapeutic, and prognostic data were analyzed.

**Results:**

A total of 68 patients were included in the study, with a median age of 55.8 years. Forty patients (58.8%) had pseudomyxoma peritonei (PMP) at the time of diagnosis. Surgical cytoreduction was used in 44 patients (65.7%), while appendectomy was performed in 23 patients (34.3%). HIPEC was used only in fifteen patients (22.4%). Gross perforation was identified in 7 cases (10.3%). While microscopically, micro-perforation or rupture was noted in 20 patients (29.4%). Adjuvant systemic treatment was used in 50% of the patients. Over a median follow-up of 18 months, recurrence occurred in 21 patients (31.8%), while five patients (7.4%) developed distant metastasis. Mortality rate was 20.5% (n=14), including five patients who died postoperatively, while nine showed tumor-related mortality. On univariate analysis, only adjuvant treatments showed statistically significant correlation with the incidence of recurrence (p=0.007).

**Conclusions:**

The higher recurrence and mortality rates observed in our cohort compared with recent literature reflect the advanced disease burden and lower utilization of HIPEC. Future work should aim to refine patient selection for HIPEC, standardize operative approaches to minimize recurrence risk, and explore the role of adjuvant systemic therapy in reducing disease progression.

**Supplementary Information:**

The online version contains supplementary material available at 10.1007/s12672-026-04833-4.

## Introduction

Appendiceal epithelial tumors are classified into mucinous and non-mucinous types based on the production of mucin [1]. Appendiceal mucinous tumors are a rare subtype of gastrointestinal tumors found in less than 1% of appendiceal specimens [2]. They are classified into serrated polyps, hyperplastic polyps, low-grade appendiceal mucinous neoplasms (LAMN), high-grade appendiceal mucinous neoplasms (HAMN), and mucinous adenocarcinomas with or without signet-ring cell carcinomas [3, 4] LAMNs are characterized by a villous or flat proliferation of mucinous epithelium with low-grade atypia, associated with the obliteration of the muscularis mucosae and lack of destructive invasion [5].

Most patients with LAMN are asymptomatic, and the diagnostic approach is usually challenging for clinicians [6, 7]. However, these tumors can mimic appendicitis and are commonly found incidentally after appendectomy for suspected appendicitis or in patients undergoing abdominal imaging or endoscopy [8]. Appendectomy with routine follow-up is often sufficient in the management of LAMNs if it is limited to the appendix with clear resection margins (R0), with no residual disease, and with no peritoneal mucin spillage [9]. However, the management of LAMN with peritoneal mucin spillage is more complex, depending on whether the mucin is cellular or acellular, as the two categories carry different prognoses after surgery [10].

The prognosis is generally good for limited LAMNs that undergo curative resection [11]. Rupture of LAMNs usually results in intraperitoneal dissemination of neoplastic cells and mucin, leading to mucinous ascites and pseudomyxoma peritonei (PMP) [8, 12]. Hence, non-localized disease carries a worse prognosis.

Given the unique and uncommon incidence of LAMN, studying its presentation, treatment, and outcomes can help highlight this disease, adding a brick to build solid evidence for establishing management guidelines.

## Methods

### Source

This is a retrospective study that included patients with pathologically proven LAMN who were presented to our center from January 2008 to December 2024. The epidemiological, clinical, therapeutic, and prognostic data of the included patients were analyzed.

### Variables

- Demographic: Age, gender, and BMI.
- Clinical: complaint, tumor size, lymph nodes, imaging, biopsy, surgical type, and approach, hyperthermic intraperitoneal chemotherapy (HIPEC), and complications.
- Pathological: type, size, and lymph node metastasis,
- Prognostic: stage, adjuvant treatment, recurrence, metastasis, and survival.

### Inclusion criteria

- Pathologically proven low-grade appendiceal mucinous neoplasm (LAMN).
- Patients aged more than 18 years.
- ECOG (Eastern Cooperative Oncology Group) Performance Status of 0–2.
- Active surgical or medical management at our center during the study period.
- Available clinical, radiological and pathological data.

### Exclusion criteria

- Appendiceal mucinous neoplasms other than LAMN (e.g., HAMN, mucinous adenocarcinoma).
- Non-confirmed pathological diagnosis.
- Completely managed patients outside our center.
- Presence of other non-colonic malignancy.
- Uncontrolled psychiatric disorders.
- Pregnant females.
- Incomplete or missing key clinical, pathological or follow-up data.

### Radiological assessment

#### Sonographic examination

Pelviabdominal ultrasound examinations were done with a conventional transabdominal technique. For all cases, the size, echogenicity, and composition were assessed. Also, Doppler imaging was done to assess the vascularity of internal soft tissue component. The presence of ascites and peritoneal deposits was also evaluated.

#### CT scans

CT scans were accomplished on a 128 multidetector CT scanner. Patients were instructed to fast for 4 6 h before imaging. Scan started from the level of the diaphragm to the symphysis pubis utilizing the following acquisition parameters: 120 kVp, 220–400 mAs, section thickness of 5 mm, window width 400, and matrix 512 × 512. Nonionic contrast medium was given at a 1.5-2 ml/kg dose with an automatic injector.

#### Pelvic-abdominal MRI examinations

Pelvic-abdominal MRI examinations were done using a 1.5-T MR scanner. Imaging started with routine sequences, including T2-weighted images (T2-WI) in the sagittal, axial, and coronal planes, as well as T1WI with and without fat suppression. Diffusion-weighted images were acquired before contrast injection with b values (0, 600, 800 s/mm2). Then, postcontrast images were gained after IV injection of Gadoterate meglumine (0.1–0.2 mmol/kg) by an automatic injector.

#### Image interpretation

All CT and MRI examinations were assessed for the important imaging features, including the lesion size (the maximum diameter) [2], presence of cystic changes [3], presence of ascites [4], nodal involvement [5], presence of peritoneal deposits, and [6] presence of PMP.

#### Surgical intervention

All surgeries were performed under general anesthesia. Laparoscopy or laparotomy was used. A midline incision was usually used for laparotomy. An assessment of the peritoneal cancer index (PCI) was made to determine operability. Multi-organ resection was performed when needed, including resection of the colon, small intestinal segments, gall bladder, pancreas, uterus, liver capsule, visceral and parietal peritoneum. CC-score was recorded for documentation of the completeness of cytoreduction. Then, HIPEC was performed if cytoreduction was feasible and the HIPEC machine and kits were available. The most administered chemotherapy was Fluorouracil with/without leucovorin. Flurouracil was given at a dose of 600 mg /m2 with a temperature of about 41.7 c over 90 min.

### Pathology review

An independent pathologist reviewed the patients’ reports for gross characteristics, including the size of the appendix, the presence of perforation and calcification, and visible mucin deposits either within the lumen or on the outer (serosal) surface. Hematoxylin-and eosin-stained slides of formalin-fixed tissue sections were acquired from the pathology lab archive and reviewed to confirm the diagnosis. Each patient’s slides were assessed for the presence or absence of lamina propria, muscularis mucosa, and lymphoid tissue. The wall of the lesion was examined for features such as calcifications, fibrosis, and micro-perforations. The maximum infiltration depth of neoplastic epithelium or acellular mucin within the appendix was documented. Additionally, peritoneal and ascetic fluid cytology reports, available for all patients, were reviewed.

#### Primary outcome

The disease-free survival.

#### Secondary outcome

The development of metastases and overall survival.

### Statistical analysis

Statistical analysis was performed using JASP software (v 0.19.3, University of Amsterdam) and Microsoft^®^ Excel^®^ for Microsoft 365 MSO (v 2311). Numerical variables were estimated using means and standard deviations for normally distributed data and medians with interquartile ranges for non-normally distributed data, respectively. Categorical variables were presented as percentages. For comparison, the Chi-square test was used for categorical variables, while Student’s t-test was used for normally distributed data, and the Mann-Whitney U test for non-normally distributed data. Kaplan-Meier survival analysis was used to evaluate time-to-event outcomes. Cox regression models were used for multivariate analysis in time-to-event outcomes. Hazard ratios and 95% confidence intervals were reported. A p-value less than 0.05 was considered significant.

## Results

**Fig. 1:**
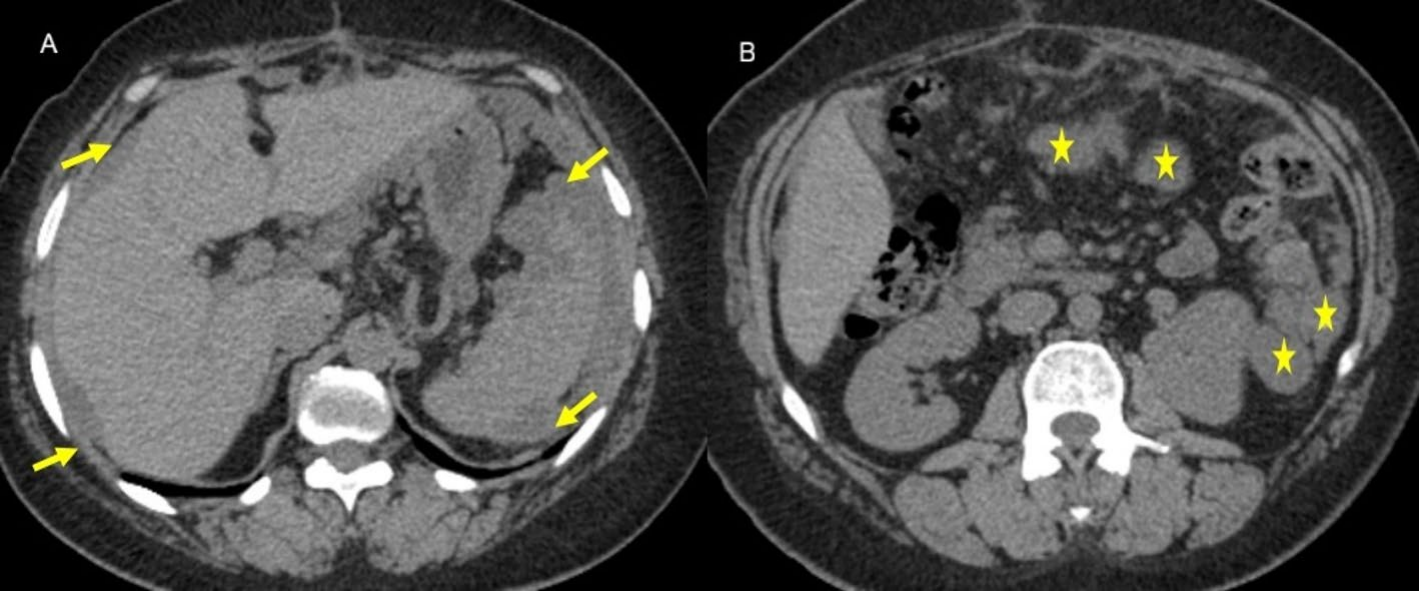
CT of a patient of LAMN with pseudomyxoma peritonei. A 47-year-old woman with pseudomyxoma peritonei and peritoneal deposits. **A** and **B** Axial CT images show a relatively high-density fluid at both hypochondrial regions, scalloping both hepatic and plenic surfaces (arrows), and multiple peritoneal deposits, the largest at the left lumbar region measures 3 cm (asterisk)

A total of 85 patients were initially evaluated for inclusion in the study. Seventeen patients were excluded. Finally, 68 patients were included in the study. The median age was 55.8 years (IQR:47.8–81), and the majority were female patients (*n* = 55, 80.9%). The median BMI was 33.9 Kg/m^2^. The most common presenting symptom was abdominal pain in 37 patients (60.7%), and the median value of CEA was 19.7 ng/ml, while the CA19-9 median value was normal (21.9 U/ml). The most used imaging modality was CT for 52 patients (76.5%) (Fig. 1), followed by MRI for 13 patients (19.7%). Forty patients (58.8%) had already developed PMP at the time of diagnosis.

Surgical treatment was performed through an open approach in 59 patients (88.1%) while laparoscopy was used in 8 patients only (11.9%). The median operative time was 262.5 min (IQR:150-307.5). Surgical cytoreduction was the most used procedure in 44 patients (65.7%), while appendectomy was performed in 23 patients (34.3%). HIPEC was used only in fifteen patients (22.4%).

Regarding the gross examination of the resected specimens, the average appendiceal diameter was 7.5 cm. A total of 49 cases (72%) showed dilatation with mucin content upon sectioning, while mucin deposits on the appendiceal serosal surface were observed in 19 cases (27.9%). Gross perforation was identified in 7 cases (10.3%), and calcifications were noted in 4 cases (5.9%).

**Fig. 2:**
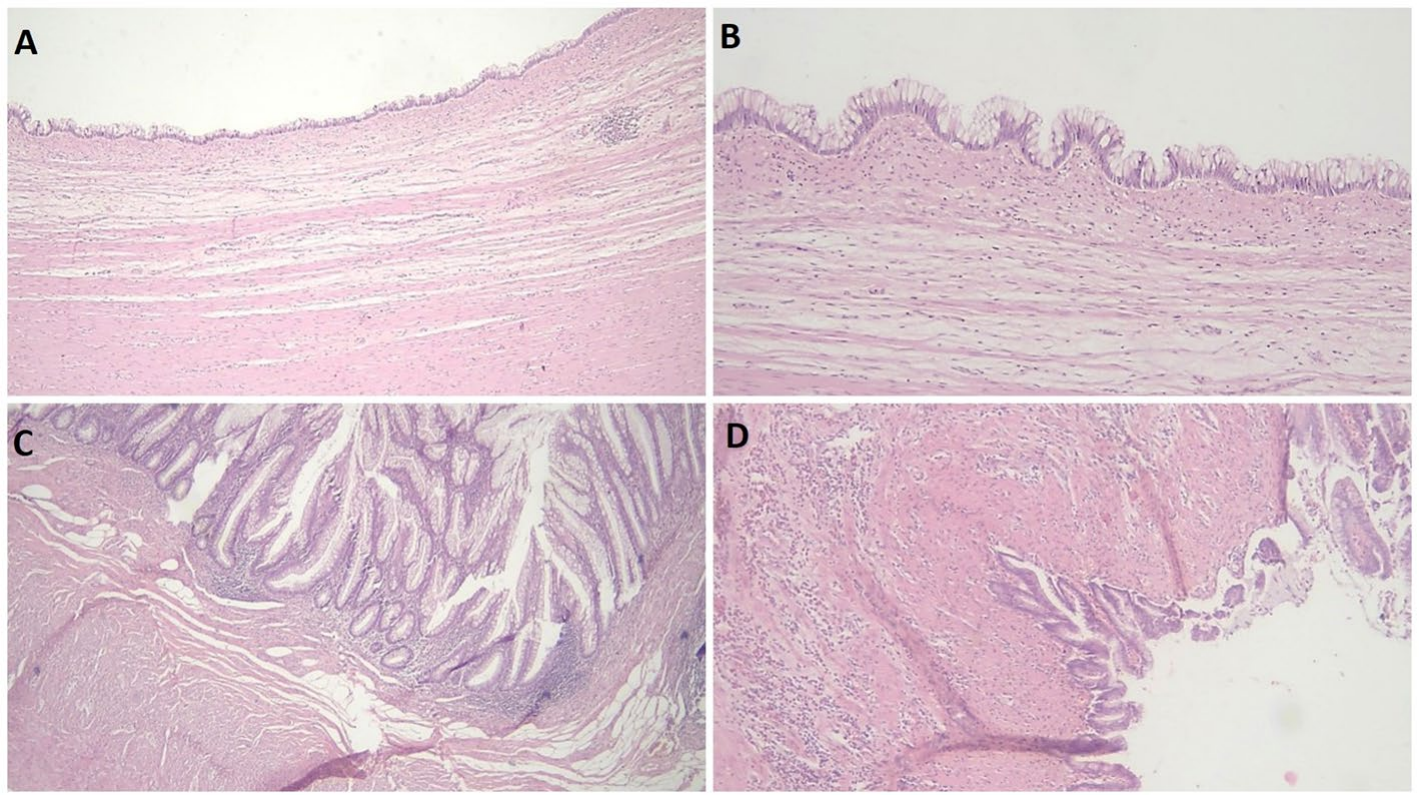
Characteristic histopathologic Features of LAMN. **A** LAMN showing a flat mucosal epithelium with loss of lamina propria, obliteration of the muscularis mucosae, underlying fibrosis, and atrophic lymphoid tissue. **B** Neoplastic mucinous epithelium with focal papillary architecture; cells display abundant apical mucin, elongated nuclei, and low-grade nuclear atypia. **C** Villous architecture of the neoplastic epithelium, with absence of lamina propria and muscularis mucosae. **D** LAMN exhibiting an undulating mucosa and pushing invasion into the underlying layers

Regarding the microscopic characteristics of the cases included in this study (as demonstrated in Fig. 2), the mucosal lining exhibited a flat, single-layered, or mildly undulating architecture composed of tall, columnar mucinous epithelium with abundant intracytoplasmic mucin and basally located, mildly atypical nuclei with pushing invasion. Focal areas displayed villous projections. The underlying appendiceal wall showed variable degrees of fibrosis, frequently accompanied by loss of the lamina propria, atrophic lymphoid tissue, and obliteration of the muscularis mucosa.

Neoplastic epithelial cells confined to the mucosa were identified in 22 cases (32.5%). In addition, deposits of neoplastic epithelial cells and/or acellular mucin were observed in the submucosa in 5 cases (7.4%), muscularis propria in 4 cases (5.8%), subserosa in 8 cases (11.7%), and serosa in 29 cases (42.6%). Appendiceal micro-perforation or rupture was noted in 20 patients (29.4%).

**Fig. 3:**
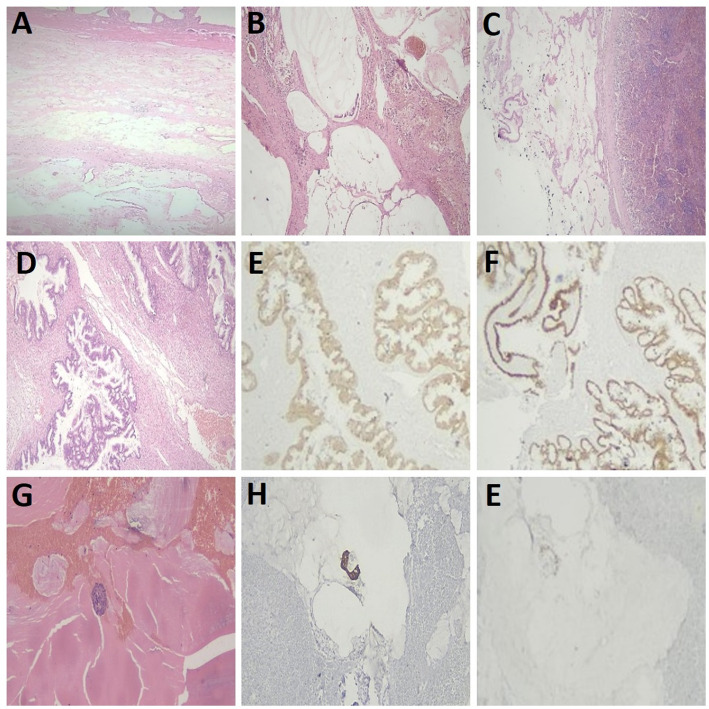
Disseminated LAMN: Histopathology, Cytology, and immunohistochemical findings. **A** LAMN with extra appendiceal mucin. **B** Cellular mucin peritoneal deposits. **C** Cellular mucin deposits on the outer surface of the spleen. **D** Ovarian involvement in a LAMN case by low-grade mucinous carcinoma; immunohistochemistry shows positive CK20 (**E**) and CDX2 (**F**). **G** Peritoneal fluid aspiration showing a few atypical cells in a mucinous background; cells express CK20 (**H**) and CDX2 (**I**)

As shown in Fig. 3, cellular mucinous deposits were present in 31(45.5%) patients, while acellular mucinous peritoneal deposits were identified in 11 patients (16.2%). Among those with cellular deposits, 17 patients (54.8%) experienced disease recurrence, and 4 patients (12.9%) developed distant metastases. In addition, out of the total cases, positive peritoneal cytology was identified in 20 patients (29.4%). Immunohistochemical analysis was performed in 23 cases, revealing negative CK7 and positive CK20 along with positive expression of caudal type homeobox 2 (CDX2) and/or Special AT-rich sequence-binding protein 2 (SATB2). This immunophenotype supports a lower gastrointestinal (colorectal/appendiceal) origin for the neoplastic epithelium.

Adjuvant systemic treatment was used in 50% of the patients, with capecitabine being the most used protocol for 25 patients (75.8% of all treated patients).

Over a median follow-up of 18 months (IQR:3–42), recurrence occurred in 21 patients (31.8%), while five patients (7.4%) developed distant metastasis. The peritoneum was the most common site for recurrence in 13 patients. Surgery was used to treat recurrence in 11 patients, while chemotherapy was used in 8 patients. The mortality rate was 20.5% (*n* = 14), including five patients who died postoperatively, while nine showed tumor-related mortality. The basic clinical, diagnostic, therapeutic, and outcomes data are summarized in Table 1.

**Table 1:** The basic clinical, diagnostic, therapeutic, and outcomes data

Variable	Number	Percent (%)
Age (years)	55.8 ((IQR:47.8–81))	
Gender Male Female	13 55	19.1 80.9
BMI (Kg/m^2^)	33.9 (IQR: 29.1–40.7)	
Presentation (*n* = 61) Accidental Abdominal pain Abdominal enlargement Vaginal bleeding Bleeding per rectum	5 37 17 1 1	8.2 60.7 29 1.6 1.6
Tumor markers CEA (ng/ml) CA 19 − 9 (U/ml)	19.7 (IQR: 5-68.7) 21.9 (IQR: 11.2–48.5)	
Imaging Imaging modality (*n* = 66) CT MRI PET-CT Tumor largest diameter (millimeters)	52 13 1 92.5 (IQR: 53.8–130)	76.5 19.7 1.5
Preoperative biopsy Yes No Biopsy type (*n* = 34) Appendectomy Core needle biopsy Fine needle aspiration Missing	34 34 5 10 14 5	50 50 14.7 29.4 41.2 14.7
Pseudomyxoma peritonei at presentation Yes No	40 28	58.8 41.2
Surgical details (*n* = 67) Operation route Open Laparoscopic Operation type Appendectomy Cytoreduction: • Right hemicolectomy • Total colectomy • Total abdominal hysterectomy & bilateral salpingoophrectomy HIPEC Yes No Operative time (minutes) Postoperative complications Yes No	59 8 23 44 5 1 1 15 52 262.5 (IQR: 150-307.5) 10 57	88.1 11.9 34.3 65.7 7.5 1.5 1.5 22.4 77.6 14.9 85.1
Pathology pT (*n* = 33) Tis T3 T4 pN (*n* = 33) Nx N0 N1 Number of harvested lymph nodes Immunohistochemistry Yes No	4 6 23 8 24 1 7 (IQR: 3–14) 28 39	12.1 18.2 69.7 24.2 72.7 3 41.8 58.2
Adjuvant treatment (*n* = 66) Yes No Type of adjuvant Protocol of adjuvant (*n* = 33) FolFOX Capecitabine Taxan-based chemotherapy	33 33 7 25 1	50 50 21.2 75.8 3
Recurrence (*n* = 66) Yes No Time to recurrence (months) Site of recurrence (*n* = 21) Anastomosis Peritoneum Omentum liver surface Spleen surface Management of recurrence (*n* = 21) Chemotherapy Surgery Follow up till progression	21 45 18 (IQR: 10.5–34.5) 1 13 3 3 1 8 11 2	31.8 68.2 4.7 61.9 14.3 14.3 4.7 38.1 52.4 9.5
Later pseudomyxoma (*n* = 62) Time till pseudomyxoma (months)	10 24 (IQR: 12–36)	16.1
Distant metastasis Yes No Time to distant metastasis (months) Site of metastasis (*n* = 6) Ovary Liver Lung	5 63 19.5 (IQR: 9.75–29.5) 1 2 2	7.4 92.6 20 40 40
Mortality Yes Postoperative Disease specific No No data	14 5 9 45 9	20.5 7.3 13.2 66.2 13.2
Follow up (months)	18 (IQR: 3–42)	

Numerical variables are presented as median & IQR

On univariate analysis, the variables were compared with outcomes (recurrence, metastasis, and mortality) (Table 2). Only adjuvant treatments showed statistically significant correlation with the incidence of recurrence (*p* = 0.007), while age, gender, BMI, markers, the use of HIPEC, operation type, and time, radiological and pathological tumor size did not show a statistically significant correlation with any of the outcomes.

**Table 2:** Univariate analysis of the variables against recurrence, metastasis, and mortality

Variable	Outcome	*P* Value
Numerical variables		
Age	Recurrence Metastasis Mortality	0.646 0.399 **0.038**
BMI	Recurrence Metastasis Mortality	0.120 0.737 0.853
CEA	Recurrence Metastasis Mortality	0.873 0.118 0.900
CA19_9	Recurrence Metastasis Mortality	0.201 0.749 0.363
Tumor’s largest diameter	Recurrence Metastasis Mortality	0.913 0.036 0.776
Operation time	Recurrence Metastasis Mortality	0.229 0.945 0.266
Categorial variables		
Gender (male vs. female)	Recurrence Metastasis Mortality	0.287 0.259 0.632
Operation type (cytoreduction vs. appendectomy)	Recurrence Metastasis Mortality	0.325 0.281 0.366
Operation approach (lap. Vs open)	Recurrence Metastasis Mortality	**0.015** 0.392 0.469
Hyperthermic intraperitoneal chemotherapy (HIPEC used or not)	Recurrence Metastasis Mortality	0.575 0.894 0.948
Adjuvant treatment (yes, no)	Recurrence Metastasis Mortality	**0.007** 0.163 0.189
Pathological T (Tis vs. T3 vs. T4)	Recurrence Metastasis Mortality	0.157 0.783 0.497

For the numerical values, Student’s t-test and Mann-Whitney test were used. For the Student t-test, the effect size is given by Cohen’s d. For the Mann-Whitney test, the effect size is given by the rank biserial correlation

**Fig. 4:**
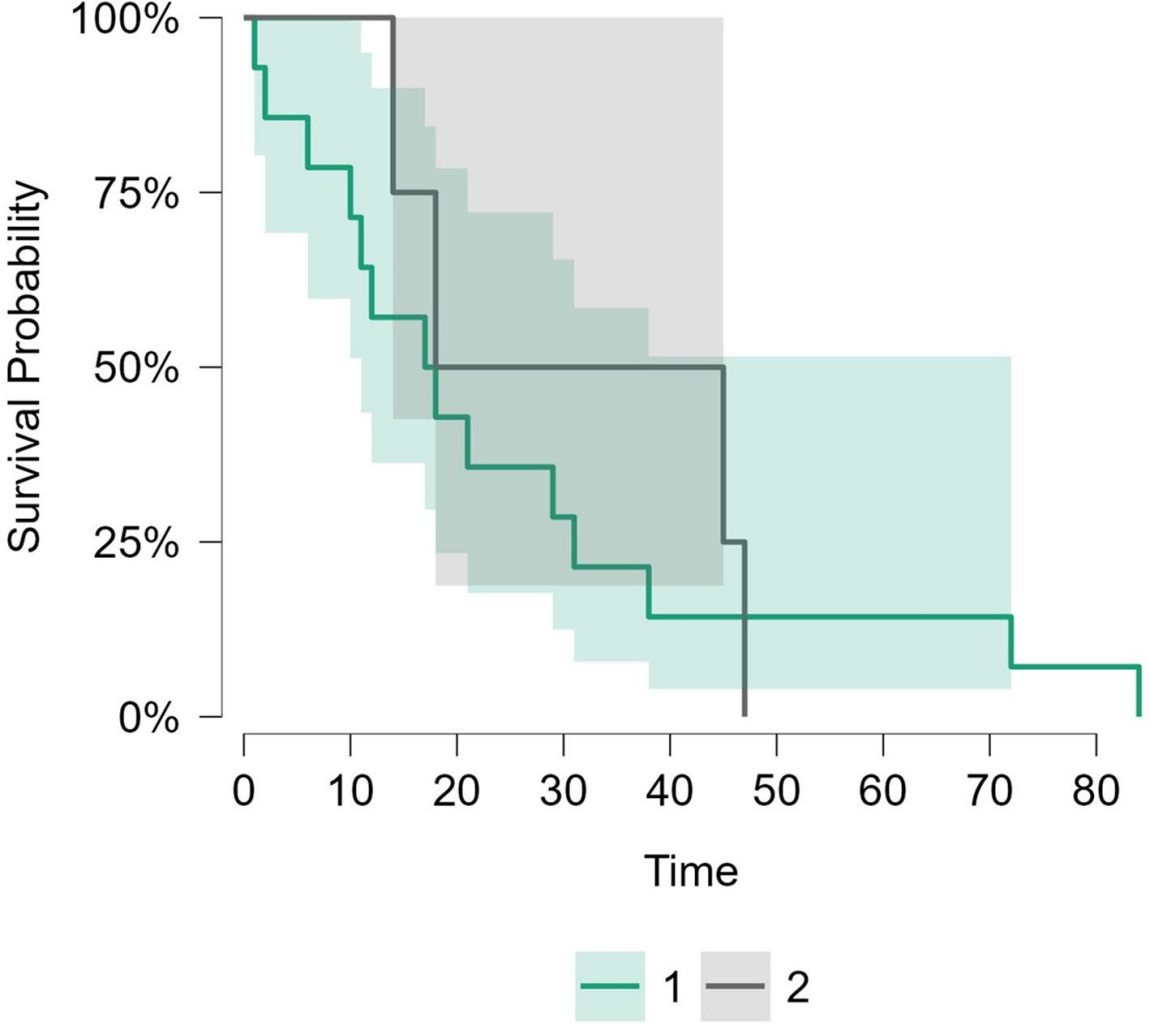
Kaplan-Meier survival analysis of the patients who received adjuvant therapy. 1 Received adjuvant therapy. 2 Did not receive adjuvant therapy

Kaplan–Meier survival analysis (Table 3) showed no significant correlation of the included variables with DFS or OAS. A multivariable Cox proportional hazards regression model was used to assess independent predictors of DFS. None of the variables showed a statistically significant independent association with DFS. Among patients who received adjuvant treatment, the median disease-free survival was 17.5 months (95% CI: 11.0–72.0), compared to 31.5 months (95% CI: 14.0—) in those who did not. The restricted mean survival time was 25.1 months for the adjuvant group and 31.0 months for the non-adjuvant group. However, it did not retain significance after adjusting for confounders (*p* = 0.628) (Fig. 4, supplementary table).

**Table 3:** Survival analysis

Variable	Outcome	*P* Value	Hazard ratio	95% CI	
				Lower	Upper
Gender	DFS OAS	0.781 0.910	2.658 0.880	0.645 0.096	10.956 8.099
Operation type	DFS OAS	0.629 0.841	0.664 0.942	0.248 0.522	1.778 1.697
Operation approach (lap. Vs open)	DFS OAS	0.423 0.139	0.564 2.440	0.136 0.749	2.336 7.945
HIPEC	DFS OAS	0.597 0.951	1.899 1.069	0.546 0.128	6.608 8.923
Adjuvant treatment	DFS OAS	0.628 0.990	1.090 1.010	0.260 0.199	4.570 5.117

## Discussion

LAMNs demonstrate a wide range of sizes, with some cases reaching notably large dimensions. In our series, the average appendiceal diameter was 7.5 cm, with a maximum length of up to 18 cm. Another study reported that LAMNs confined to the appendix ranged in size from 3.8 cm to 10.6 cm, with an average diameter of approximately 6.6 cm [13]. A case report documented a giant LAMN measuring approximately 16.5 cm in length and 7 cm in diameter [14]. These findings highlight the importance of considering LAMNs in differential diagnoses, even in cases where the appendix exhibits significant enlargement.

Approximately 72% of LAMN cases in our study demonstrated a mucin-filled lumen, and 5% showed gross calcifications. These findings are consistent with those reported in previous studies [15]. In our study, 27.9% of cases showed mucin deposits on the appendiceal serosal surface. Gross full-thickness perforations were identified in 10.3% of cases, while micro-perforations were observed in 29.4%. Several studies [16, 17] have reported a higher incidence of serosal mucin deposits (34-39.9%), compared to overt perforation in LAMN (19.3–22.6%). This discrepancy may be attributed to underreporting of gross perforations due to limited sampling or to the possibility of mucin tracking through a fibrotic or thinned appendiceal wall, or via appendiceal diverticula. Many such cases are thought to result from microperforations. Understanding this relationship is clinically significant, as the presence of mucin on the serosal surface escaping through undetected micro-perforations may increase the risk of peritoneal dissemination.

Consistent with previous reports in the literature [18, 19] LAMNs in our series exhibited flat or papillary mucinous epithelium with low-grade cytologic dysplasia. Additionally, these neoplasms demonstrated characteristic changes in the appendiceal wall, including effacement of the lamina propria, atrophic lymphoid tissue, and obliteration of the muscularis mucosa within a variably fibrotic wall.

In the current study, tumors confined to the mucosa ± acellular mucin or neoplastic epithelium in the submucosa or muscularis propria without destructive invasion (Tis) were observed in 45.5% of cases. Acellular mucin or neoplastic epithelium extending into the subserosa (T3) was detected in 11.7%, while tumor or mucin on the serosal surface (T4) was identified in 42.6% of cases. Comparatively, many studies [20, 21] reported higher proportions of Tis cases (60–64%). In contrast, other studies [16, 17, 22] found that most of their cases (57%) were advanced-stage tumors (T4). The variations in the histopathological staging of LAMN across these studies, including the current one, can be attributed to several key factors, such as earlier incidental detection in some cohorts versus advanced presentations in others, which can significantly influence staging outcomes. Differences in sample sizes also further affect comparability. Additionally, variation in the pathological assessment of tumor invasion and mucin dissemination, especially in borderline cases, introduces additional variability.

In our study, cytological analysis of ascitic fluid or peritoneal washings was available for a subset of cases, with positive cytology detected in 29.4%. Additionally, 16.2% of cases exhibited peritoneal lesions with acellular mucin, while 45.5% demonstrated cellular mucinous deposits. Notably, approximately 54.8% of patients with cellular deposits experienced disease recurrence, and 12.9% developed metastases. Among studies examining LAMN associated with PMP and peritoneal deposits, the reported proportions of acellular and cellular mucinous deposits vary considerably. For example, a study by Roxburgh et al. reported cellular mucinous peritoneal deposits in 39.6% of patients, versus 60.4% with acellular deposits [23]. In contrast, another study found a higher prevalence of cellular mucinous peritoneal deposits (58.7% vs. 41.3%) [24]. These findings emphasize the crucial role of differentiating cellular from acellular mucinous deposits and highlight the prognostic significance of cellular peritoneal involvement.

In our study, most patients (98.5%) underwent surgical intervention, with cytoreduction procedures representing the largest proportion (65.7%), followed by appendectomy (34.3%). Appendectomy is reported to demonstrate favorable short-term outcomes and overall survival (OS) in cases where the tumor was unruptured and surgical margins were negative [25]. Complete cytoreductive surgery (CRS) followed by HIPEC remains the standard of care for PMP [26].

One of the most significant findings in our study is the percentage of patients who received adjuvant therapy (33 patients = 50%). While most of them received oral Capecitabine (25 patients = 75.8%) and only one quarter received systemic chemotherapy, the percentage is still very high when compared to the recent literature that reported percentages ranging from 10 to 12% [25, 27]. This can be attributed to three points. Firstly, the large percentage of patients with T4 tumors and with advanced tumors who underwent cytoreductive surgery. Secondly, the large time period of the study, starting from 2008, with heterogeneous treatment protocols. Thirdly, the limited number of patients who underwent HIPEC due to financial restrictions and the delayed introduction of the HIPEC machine to our center in 2016.

Our cohort exhibited a recurrence rate of 31.8%, which is notably higher than the most recent LAMN series reporting recurrence rates ranging from 3% to 12.5% [25, 28, 29]. However, this rate was slightly lower than the 36% recurrence reported in subgroups with peritoneal dissemination (PD) [30]. A likely explanation is that 58.8% of our patients presented with PMP at diagnosis, a known adverse prognostic factor. This highlights the fact that a more advanced presentation represents an additional challenge in low-resource settings and low health literacy. Importantly, laparoscopic resection was linked to higher recurrence compared with open surgery(p-value = 0.015). Although some recent studies did not identify surgical approach as an independent predictor of recurrence or survival, the increased recurrence in our laparoscopic cases may reflect the higher disease burden in these patients and the corresponding elevated risk of mucin spillage during minimally invasive procedures [25]. 

The mortality rate in our cohort was 20.5% at a median follow-up of 18 months, which is markedly higher than in recent publications. Multiple series report 5-year OS between 83.3% and 96.8%, and even higher survival (~ 97%) following appendectomy in non-perforated cases [25, 29, 31]. This discrepancy reflects differences in baseline disease severity, as well as the high proportion of PMP at presentation in our study. Advanced age was significantly associated with mortality, in line with prior work showing poorer outcomes in patients over 60 years [25]. Although adjuvant chemotherapy was significantly linked with more loco-regional recurrence in our cohort (p value = 0.007), it did not affect survival, partially consistent with recent registry data reporting a significantly negative impact of adjuvant chemotherapy on OS in LAMN [25]. This is likely because therapy is typically administered to higher-risk patients, and not a direct negative effect of treatment, as evidenced by the loss of significance in multivariate analysis (*p* = 0.628). In contrast to some reports linking elevated preoperative CEA levels to poorer survival, CEA and CA-19-9 were not significant prognostic indicators in our population, possibly due to sample size limitations and short follow-up time [32]. 

In patients with concurrent PMP, HIPEC was performed in only 22.4% of cases and did not significantly affect survival or recurrence. In contrast, other studies have demonstrated improved outcomes with CRS + HIPEC, including an 18-month OS approaching 95% and a 5-year OS of 70–75%, as well as 5-year OS rates exceeding 80% in perforated LAMN [32, 33]. The lack of survival benefit in our series may reflect selection bias toward more advanced disease or lower utilization of HIPEC that is not aligned with the percentage of PMP, unlike in this recently published study [25]. 

Progression to PMP occurred in 16.1% of patients after treatment, with a median interval of 24 months, which is comparable to previously reported progression rates of ~ 20% at a median of 12 months following appendectomy [34]. These findings underscore the importance of prolonged surveillance, particularly in patients with risk factors such as perforation, mucinous spillage, or peritoneal involvement at presentation.

This study has limitations; firstly, it is retrospective, which opens the door to selection bias and data heterogeneity. Secondly, the limited availability of HIPEC in the management of patients with PMP due to financial and logistical obstacles. Thirdly, we fully acknowledge that 5-FU is not a conventional HIPEC agent and that the evidence base is limited. For this reason, we clearly state that our findings should be interpreted as observational and hypothesis-generating. Fourthly, the small sample size and fifthly, the short available follow-up period, which makes the findings less strong than the studies with longer follow-up periods. Sixthly, not reporting the PCI and CC- score for PMP patients due to data insufficiency.

## Conclusions

Overall, our results highlight the prognostic impact of LAMN disease stage at presentation and the cellularity of peritoneal deposits. In the resource-limited setting where LAMN presents with higher rates of PMP, the outcomes of LAMN are worse than reported in the literature. Standardized operative approaches and appropriate surveillance are required to minimize recurrence risk and improve outcomes.

## Supplementary Information

Below is the link to the electronic supplementary material.

Supplementary Material 1.

## Data Availability

All the clinical, radiological & pathological data used in this manuscript are available in the Mansoura University medical system (Ibn Sina Hospital management system). [http://srv137.mans.edu.eg/mus/newSystem/](http:/srv137.mans.edu.eg/mus/newSystem).
