# Supplementary material for: The clinical and pathological features of low-grade appendiceal mucinous neoplasm (LAMN)
Source: Discov Oncol. 2026 Apr 2;17:551. doi: 10.1007/s12672-026-04833-4 (PMC13066059; doi:10.1007/s12672-026-04833-4)
Supplement: Supplementary file 1 — Supplementary Material 1. [file 12672_2026_4833_MOESM1_ESM.docx]

**Non-parametric Survival Analysis**

| *Kaplan-Meier Summary Table* | | | | | | | | | | | | | | | |
| --- | --- | --- | --- | --- | --- | --- | --- | --- | --- | --- | --- | --- | --- | --- | --- |
|  | | | | | | | | | | | | 95% CI | | | |
| Strata | | N | | Events | | Restricted Mean | | Standard Error | | Median Survival | | Lower | | Upper | |
| Adjuvant treatment=yes |  | 14 |  | 14 |  | 25.143 |  | 6.427 |  | 17.500 |  | 11.000 |  | 72.000 |  |
| Adjuvant treatment=no |  | 4 |  | 4 |  | 31.000 |  | 7.542 |  | 31.500 |  | 14.000 |  |  |  |
|  | | | | | | | | | | | | | | | |
| *Note.*  50 observations omitted due to missing values. | | | | | | | | | | | | | | | |

**Semi-parametric Survival Analysis**

| *Cox Proportional Hazards Estimates Table* | | | | | | | | | | | | | | | |
| --- | --- | --- | --- | --- | --- | --- | --- | --- | --- | --- | --- | --- | --- | --- | --- |
|  | | | | | | | | 95% CI | | | |  | | | |
| Model | |  | | Estimate | | Standard Error | | Lower | | Upper | | z | | p | |
| H₁ |  | Adjuvant treatment |  | -0.273 |  | 0.583 |  | -1.416 |  | 0.870 |  | -0.468 |  | 0.640 |  |
|  | | | | | | | | | | | | | | | |

| *Hazard Ratios Estimates Table* | | | | | | | | | |
| --- | --- | --- | --- | --- | --- | --- | --- | --- | --- |
|  | | | | | | 95% CI | | | |
| Model | |  | | Hazard Ratio | | Lower | | Upper | |
| H₁ |  | Adjuvant treatment |  | 0.761 |  | 0.243 |  | 2.387 |  |
|  | | | | | | | | | |
